# HIV and Aging: Overcoming Challenges in Existing HIV Guidelines to Provide Patient-Centered Care for Older People with HIV

**DOI:** 10.3390/pathogens10101332

**Published:** 2021-10-15

**Authors:** Aroonsiri Sangarlangkarn, Yuji Yamada, Fred C. Ko

**Affiliations:** 1 Department of Medicine, Temple University, Philadelphia, PA 19122, USA; 2 Brookdale Department of Geriatrics and Palliative Medicine, Icahn School of Medicine at Mount Sinai, Manhattan, NY 10029, USA; yuji.yamada@mssm.edu (Y.Y.); fred.ko@mssm.edu (F.C.K.)

**Keywords:** guidelines, HIV, geriatrics, function, cognition, frailty

## Abstract

With advances in antiretroviral therapy and subsequent increase in life expectancy, People with HIV (PWH) now experience multiple geriatric syndromes in the setting of advanced aging and increased multimorbidity. HIV clinicians bear the responsibility of delivering geriatric care to this vulnerable population, despite limited geriatric medicine training and limited support from HIV service networks that were not traditionally designed to care for an aging population. Although HIV clinicians reported formal guidelines specific to older PWH to be among the most helpful interventions, current HIV guidelines present multiple issues in their applicability to the care of older PWH, including multifactorial nature of conditions in older adults, difficulty measuring patient-centered outcomes, lack of representation of older PWH in clinical trials, limited guidelines addressing geriatric syndromes, and the use of chronological age as criteria for inclusion despite advanced aging in PWH. Understanding that updated guidelines addressing above challenges may take many years to develop, we offer strategies on the application of current guidelines, including using baseline attributes, time to benefit, and the Geriatrics 5M model to aid in shared decision making and improve outcomes among older PWH.

## 1. Introduction

With the invention of antiretroviral therapy, life expectancy among People with HIV (PWH) has significantly increased. In 2018, 51% of people in the United States with HIV were aged 50 years or older, with 1 in 6 new HIV diagnoses occurring among this age group [1]. It is projected that by 2020, 21% of PWH globally would be aged 50 years and older [2].

With increased life expectancy, older PWH are at risk of advanced aging [3,4], increased multimorbidity [5], as well as geriatric syndromes [6]. However, older PWH lack adequate access to geriatric care due to worldwide shortages of geriatricians, as well as the fact that geriatricians are usually not trained in the care of PWH during geriatrics fellowship. Consequently, HIV clinicians bear the responsibility of providing geriatric care to this population, despite limited geriatrics training and limited support from HIV service networks that were not traditionally designed for an aging population [7].

To identify strategies to overcome above challenges, a survey found that, according to HIV clinicians, developing formal guidelines specific to older PWH is among the most helpful interventions [8]. However, existing HIV guidelines present multiple issues in their applicability to the care of older PWH.

## 2. Challenges Pertaining to Existing HIV Guidelines in the Care of Older PWH

Despite increasing numbers of older PWH, existing HIV guidelines present multiple challenges in the care of this population. These limitations are secondary to various issues associated with clinical guideline development for older adults, some of which parallel those encountered in guidelines for non-HIV related conditions [9]. These issues include:

### 2.1. Multifactorial Nature of Conditions

In older adults, a disease is often contributed by multiple risk factors instead of a single cause. This means that an effective and practical guideline will need to evaluate multifactorial interventions as well as measures of multiple disease-related outcomes. As an example, a guideline on frailty syndrome may explore multiple frailty-related outcomes such as falls, physical function, ambulation, and quality of life. With such complexity related to the multifactorial nature of geriatric conditions, clinical studies in older adults may be difficult to standardize and synthesize into systematic reviews and meta-analyses that can be used to inform existing guidelines in older PWH. 

Additionally, due to the multifactorial nature of conditions, disease-centric guidelines may recommend treatments without considering side effects that may worsen another disease, a complexity commonly encountered among older patients with multimorbidity [10]. For example, a hypertension guideline advocating for tight blood pressure control may clash with another guideline on fall prevention, in which certain blood pressure reducing medications and stringent control may have an unintended consequence of increasing fall risks.

### 2.2. Measurement Issues

Existing guidelines often focus on traditional, easy-to-measure clinical outcomes such as mortality or hospital admissions. However, the most relevant health outcomes for older PWH may include entities that are not measurable as discreet events or easily comparable/reproducible across studies, such as physical function, cognition, or quality of life.

### 2.3. Lack of Representation in Clinical Trials

In the uninfected, the sickest, most vulnerable older adults are often excluded from randomized controlled trials, even though this is the population that is most challenging to care for. This underrepresentation in clinical trial participation poses a barrier in the development of guidelines optimized for the care of older adults. In PWH, this issue is both present and compounded by the fact that older PWH is a relatively new and emerging population, and their limited representation in existing HIV guidelines contributed to the paucity of evidence and limited applicability to older PWH. 

Additionally, existing HIV guidelines are curtailed by challenges unique to older PWH, including:

### 2.4. Determining Which Patients to Screen and Apply Guidelines to

Many existing guidelines focus on chronological age as a criterion to initiate screening as it is easily measurable. However, multiple studies have shown that chronological age is poorly correlated with the physiologic age of the patient [11,12]. This issue is compounded among older PWH, as they may experience advanced aging [3,4,13,14], rendering chronological age even more inaccurate in this population.

### 2.5. Limited Guidelines Addressing Geriatric Syndromes

Many current HIV guidelines do not provide guidance on important geriatric issues such as falls, frailty, cognitive impairment, or advance care planning [15,16,17]. We noted that, although the European AIDS Clinical Society guideline [18] and the British HIV Association [19] addressed a few geriatric syndromes, it did not provide specific criteria on which patients should be included in said guidelines, making its application in older PWH challenging.

## 3. Strategies on Applying Existing Guidelines to Older PWH

Given the understanding that updated HIV guidelines addressing the above challenges will likely take many years to develop, we offered the following strategies on the application of current guidelines to improve their relevance and effectiveness among older PWH (Table 1).

### 3.1. Use Frailty or Cognitive and Functional Status to Inform Shared Decision-Making on Treatment Goals/Interventions

Coupling important baseline measurements with chronological age cutoffs can help clinicians determine which patients would be able to tolerate and therefore benefit from interventions recommended by guidelines. Important baseline domains that may limit the benefits or feasibility of interventions include poor physical function, cognition, and frailty status.

For example, the American Diabetes Association (ADA) recommends consideration of function and cognition when determining hemoglobin A1c (HbA1C) treatment goals in older adults [28]. For moderately ill patients (e.g., 2+ deficits in instrumental activities of daily living (IADL) or mild-to-moderate cognitive impairment), a reasonable HbA1C goal is <8.0% compared to <7.0–7.5% in healthy older adults with intact functional and cognitive status. For severely ill patients (e.g., 2+ deficits in activities of daily living (ADL) or moderate-to-severe cognitive impairment), ADA recommends avoiding reliance on HbA1C as a goal altogether and aiming instead to avoid symptoms from hypoglycemia or hyperglycemia. Of note, the ADA also uses places of dwelling to modify treatment goals and considers severely ill patients as those residing in a long-term care facility (vs. in the community). Although there is no existing literature to guide the application of such function or cognition modification to current HIV guidelines, the example from the ADA HbA1C recommendation may provide guidance in PWH. Clinicians should screen PWH for baseline functional and cognitive status, and those with 2+ deficits in IADL or mild-to-moderate cognitive impairment should consider a shared decision-making discussion to determine if more relaxed treatment goals may be desired. Clinicians can take a graded approach in determining new goals by first setting a treatment target close to the patient’s current disease parameters, then moving closer in a stepwise approach towards guideline goals once prior targets have been reached and no complications or side effects have occurred. For example, a patient with a blood pressure of 150/90 may first aim to reach a target of 140/90, then continue in a stepwise approach towards a guideline goal of 120/80, stopping only when she experiences adverse effects from the treatment. 

Prior studies have recommended quick, simple tools for use by HIV clinicians to assess function and cognition [22], including the mini-cog or Montreal Cognitive Assessment (MoCA) for cognition, and the Timed Get-Up-and-Go (TUG) test or ADL/IADL for function. An article by Sangarlangkarn et al. provided guidance on how best to implement such screenings in a busy HIV practice [22].

In the same way that oncologists routinely evaluate risk of treatment toxicity using models such as Chemotherapy Risk Assessment Scale for High-Age Patients (CRASH) [29], HIV clinicians should take into account the risk of adverse events from treatments they plan to prescribe based on guidelines. Frailty, as a baseline attribute, can be used to determine complication risks related to interventions when weighing risks vs. benefits. For example, prior studies have used frailty to determine risks of mortality or complications after various surgeries and cancer treatments [30,31,32]. Although extrapolation from data in the uninfected is likely necessary, HIV clinicians could utilize frailty to determine risks of adverse events for the intervention in question and use these data to guide shared decision making with patients.

In PWH, frailty can be readily determined using the Veterans Aging Cohort Study the (VACS) index, with the cutoff of >18 denoting frailty [27]. For ease of use, the VACS index utilizes commonly measured clinical laboratory values in PWH and can be easily accessed on MDCalc (Table 1).

### 3.2. Take Time to Benefit into Account

To overcome limitations related to chronological age cutoffs, considering time to benefit can help clinicians determine whom to screen, and more importantly, which patients may not benefit from screening and stringent adherence to current guidelines. The ADA guidelines for HbA1c in older adults used multiple outcomes related to time to benefit to relax treatment goals, including utilizing higher number and severity of comorbidities [28] as a proxy for life expectancy. In PWH, prognostication of life expectancy is challenging for multiple reasons. First, older PWH, as an emerging population, are rarely included in existing prognostic indices. Second, the advent of new HIV therapies and rapidly evolving knowledge base on older PWH may also complicate prognostication [33]. The VACS index can be used to determine all-cause 5-year mortality risk in PWH if there are no other diagnoses driving prognostication. However, it should be noted that the VACS index was validated in the US and its application may be limited, especially to other countries with limited resources.

Once life expectancy is determined, it can be used to guide shared decision making to determine appropriate care goals. In early-stage disease with long life expectancy, curative goals may be realistic. On the other hand, in late-stage disease with limited life expectancy, the goals may be to limit distressing symptoms, improve quality of life, and prolong survival [29]. 

### 3.3. Eliciting Patient Preferences When Guidelines Clash

When disease-centric guidelines clash in the setting of multimorbidity, clinicians may utilize the “Geriatrics 5M” model [34] to guide shared decision making, focusing on the “matters most to me” component to elicit patient’s own goals and care preferences. For example, in a multimorbid patient with hypertension and falls where treatment for one condition may worsen the other, clinicians may weigh the patient’s various desired goals and aversions to related side effects to guide medical decisions. Goals in this example may include reduced risk of stroke or avoidance of hip fractures, while aversions to related side effects may include limiting orthostasis/dizziness or reducing pill burden. For an active community-dwelling older adult who fears debilitating strokes and becoming a burden to his family, a tight blood pressure goal may be desired. On the other hand, for a bedbound, multimorbid patient in a nursing home who prioritizes limiting pill burden, a liberal blood pressure goal may be pursued instead. 

## 4. Future Directions and Conclusion

Leipzig et al. outlined methods to address deficits in guidelines in the uninfected older adults related to multifactorial nature of conditions, measurement issues, and lack of representation in clinical trials [9]. These strategies could be applied to guidelines for older PWH, although their application to resource-poor settings may be limited since the studies mentioned in this article were mostly conducted in western countries. Additionally, future research should aim to provide guidelines addressing geriatric syndromes specifically in PWH and enroll older PWH across a representative spectrum of function and cognition in clinical trials. Understanding that PWH experience advanced aging, guidelines should shift away from chronological age criteria towards physiologic age criteria using baseline function, cognition, or frailty measures. Moreover, future studies need to determine which tools accurately reflect physiologic age in PWH, ideally finding quick, simple tools that can predict multiple outcomes of interest (such as the VACS index, a single calculator that can predict function, frailty, and mortality). In the meantime, clinicians may use baseline attributes, time to benefit, and the Geriatrics 5M model to inform existing guidelines to improve outcomes and patient satisfaction among older PWH. 

## Figures and Tables

**Table 1:** Challenges pertaining to existing HIV guidelines and corresponding strategies.

Guideline Issues and Corresponding Strategies	Recommended Tools	Domains Addressed	Tool Details
***Chronological age***			
Use frailty or cognitive/functional status to inform shared decision-making on goals/interventions	Montreal Cognitive Assessment (MoCA)	Cognition	- Utilized in prior research in People with HIV (PWH) [20,21]; - Can screen for cognitive impairment of all causes (HIV-associated neurocognitive disorders as well as other causes) [22].
	Mini-cog	Cognition	- 3-word recall coupled with clock-drawing test: 3/3 recall = normal; 1 − 2/3 recall + normal clock = normal; 1 − 2/3 recall + abnormal clock = impaired; 0/3 recall = impaired.
	Timed get-up-and-go (TUG) test	Function	- Utilized in prior research in PWH [23,24]; - Explore multiple components of mobility (gait speed, balance, and proximal muscle strength); - Correlate with function and more formal tests on balance and gait speed; - Patient is timed while he/she rises from a chair, walks 3 m, turns, walks back, and sits down again; - At risk of falling if ≥12 s [25].
	Activities of daily living (ADL) and instrumental activities of daily living (IADL)	Function	- Utilized in prior research in PWH [20,26]; - Minimal training required; - Practical, focus on daily task deficits that can guide interventions and directly improve quality of life [22]; **ADL**: bathing, dressing, grooming, toileting, transferring, and eating; **IADL**: cooking, shopping, managing medications, using the phone, doing housework, doing laundry, driving or using public transportation, and managing finances.
	Residing in a long-term care facility vs. the community	Function Cognition	- Used by the American Diabetes Association to guide A1c goal
	Veterans Aging Cohort Study the (VACS) index	Frailty	- Developed specifically for PWH; - Utilizes commonly drawn lab values in PWH; - Easily accessible on MDCalc (https://www.mdcalc.com/veterans-aging-cohort-study-vacs-index) (accessed on 9 September 2021) - At risk of frailty if >18 [27].
Take time to benefit into account	Higher number and severity of comorbidities	Mortality	- Used by the American Diabetes Association to guide A1c goal
	Veterans Aging Cohort Study the (VACS) index	Mortality	- Provide all-cause 5-year mortality risk
***Multifactorial nature of conditions***			
Elicit patient preferences when guidelines clash	Geriatric 5M model	Multimorbidity	- Explore “Matters Most to Me” component; - Weigh patient’s desired goals and aversions to related side effects to guide decisions.

## Data Availability

Not applicable.
